# TMS-evoked EEG potentials demonstrate altered cortical excitability in migraine with aura

**DOI:** 10.1007/s10548-023-00943-2

**Published:** 2023-02-13

**Authors:** Robert M. Helling, Matthijs J. L. Perenboom, Prisca R. Bauer, Johannes A. Carpay, Josemir W. Sander, Michel D. Ferrari, Gerhard H. Visser, Else A. Tolner

**Affiliations:** 1 Stichting Epilepsie Instellingen Nederland (SEIN), Achterweg 5, 2103 SW Heemstede, The Netherlands; 2 Department of Neurology, Leiden University Medical Center, Albinusdreef 2, 2333 ZA Leiden, The Netherlands; 3 Department of Neurology, Tergooi Hospitals, Van Riebeeckweg 212, 1213 XZ Hilversum, The Netherlands; 4 Department of Clinical and Experimental Epilepsy, UCL Queen Square Institute of Neurology, Queen Square, WC1N 3BG London, UK; 5 Department of Human Genetics, Leiden University Medical Centre, Postal Zone S4-P, PO Box 9600, Leiden, The Netherlands; 6 Department of Psychosomatic Medicine and Psychotherapy, Faculty of Medicine, University of Freiburg, Hauptstraße 8, 79104 Freiburg, Germany

**Keywords:** TMS evoked potential, Electroencephalography, Phase clustering, Cortical excitability, Migraine pathogenesis

## Abstract

Migraine is associated with altered sensory processing, that may be evident as changes in cortical responsivity due to altered excitability, especially in migraine with aura. Cortical excitability can be directly assessed by combining transcranial magnetic stimulation with electroencephalography (TMS-EEG). We measured TMS evoked potential (TEP) amplitude and response consistency as these measures have been linked to cortical excitability but were not yet reported in migraine.

We recorded 64-channel EEG during single-pulse TMS on the vertex interictally in 10 people with migraine with aura and 10 healthy controls matched for age, sex and resting motor threshold. On average 160 pulses around resting motor threshold were delivered through a circular coil in clockwise and counterclockwise direction. Trial-averaged TEP responses, frequency spectra and phase clustering (over the entire scalp as well as in frontal, central and occipital midline electrode clusters) were compared between groups, including comparison to sham-stimulation evoked responses.

Migraine and control groups had a similar distribution of TEP waveforms over the scalp. In migraine with aura, TEP responses showed reduced amplitude around the frontal and occipital N100 peaks. For the migraine and control groups, responses over the scalp were affected by current direction for the primary motor cortex, somatosensory cortex and sensory association areas, but not for frontal, central or occipital midline clusters.

This study provides evidence of altered TEP responses in-between attacks in migraine with aura. Decreased TEP responses around the N100 peak may be indicative of reduced cortical GABA-mediated inhibition and expand observations on enhanced cortical excitability from earlier migraine studies using more indirect measurements.

**Supplementary Information:**

The online version contains supplementary material available at 10.1007/s10548-023-00943-2.

## Introduction

Migraine is a brain disease characterized by recurring attacks of severe headaches, accompanied by other symptoms like nausea, vomiting and sensitivity to light and sound (Goadsby et al. 2017). Visual aura before the headache phase, experienced by about one third of people with migraine, is a transient focal symptom likely due to cortical spreading depolarization in the visual cortex (Ferrari et al. 2015). People with migraine report increased visual sensitivity between and during attacks compared to healthy controls (Bigal et al. 2006; Perenboom et al. 2018), which appears most prominent in those with visual aura symptoms (Cucchiara et al. 2015). Altered visual cortex responsivity (Coppola et al. 2007), that could be caused by changes in cortical network excitability may explain these symptoms. However, hyperexcitability (Aurora and Wilkinson 2007; Datta et al. 2013) and hypoexcitability have both been suggested as underlying mechanism (Coppola et al. 2007), largely based on indirect measures of cortical excitability.

Transcranial magnetic stimulation (TMS) has been one of the methods used to study cortical excitability in migraine, using subjective or indirect readouts (Magis et al. 2013). Magnetophosphene induction, by applying TMS over the occipital cortex while registering the reported threshold of perceived visual responses, is a direct but subjective measure of visual cortex excitability (Salminen-Vaparanta et al. 2014). A meta-analysis suggested decreased phosphene thresholds in migraine with and without aura compared to controls when a large circular coil was used. More localized stimulation using a figure-of-eight coil resulted in increased phosphene prevalence in subjects with aura, and not in those without aura or controls (Brigo et al. 2013). Studies on motor cortex excitability have used the muscle response to single pulse TMS as indirect readout by determining a resting motor threshold (RMT). This threshold does not reflect cortical excitability exclusively, as subcortical pathway excitability will also affect muscle responses (Bestmann and Krakauer 2015). Using this method, no changes were demonstrated between migraine with or without aura in-between attacks and controls (Magis et al. 2013). Stimulus response curves of the motor response recorded by varying stimulation intensity showed contradictory patterns in migraine as well, with indications of motor cortex hyperexcitability at high stimulus intensities (Khedr et al. 2006; Cosentino et al. 2011). Motor responses to short-burst repetitive TMS differed over the migraine cycle for migraine with and without aura, which relates TMS-induced measures to cyclic changes in cortical excitability (Cosentino et al. 2014).

Advances in electroencephalography (EEG) amplifier technology allow direct recordings of the cortical network response to TMS (Ilmoniemi and Kičić 2010). Using TMS-EEG, magnetically evoked cortical responses can be evaluated as direct and objective markers of cortical responsivity, and provide information on changes in network excitation or inhibition (Chung et al. 2015). Single pulse stimulation at one location generates responses measurable over the entire scalp, enabling comparison of cortical excitability across cerebral regions (Komssi et al. 2004). The TMS-evoked potential (TEP) follows a specific pattern, of which peak amplitudes are altered by neuroactive drugs that modulate excitatory of inhibitory neurotransmission (Premoli et al. 2014; Ziemann et al. 2015). TEP amplitudes are also affected in conditions such as epilepsy and schizophrenia in which altered cortical excitability is implicated (Farzan et al. 2010; ter Braack et al. 2016). TEPs, however, have not yet been assessed in the context of migraine. In addition to amplitude characteristics, the phase of frequency components in evoked potentials (Lopes da Silva 2006) and ongoing EEG (Meisel et al. 2015) also contains relevant information on cortical excitability. Occipital phase clustering of visually evoked responses between repetitions is predictive of a photoparoxysmal response in photosensitive epilepsy (Parra et al. 2003), suggesting a relation between consistency of phase responses across stimulation trials and excitability levels.

We aimed to assess possible alterations in cortical excitability directly using TMS-EEG in subjects with migraine with aura (in-between migraine attacks) and controls. Using a circular TMS coil, we induced broad, scalp-wide activation thus not limiting the study to a predefined local stimulation site. The combination with EEG allowed us to explore local alterations in cortical excitability over the whole scalp based on local changes in TEP responses as direct measure of cortical excitability. We compared TEPs over the entire scalp to ascertain the distribution, amplitude and phase characteristics of response patterns at frontal, central and occipital electrode clusters along the midline. These readouts could provide objective parameters on cortical excitability and allow identification of migraine-specific changes in excitability across cerebral regions including the visual cortex.

## Materials and Methods

### Participants

Participants (aged 18 or over) were recruited locally through digital and paper adverts and through the LUMINA study population of the Leiden University Medical Centre (van Oosterhout et al. 2011). Matching controls were selected from a cohort of 38 healthy controls described elsewhere (Bauer et al. 2019). Migraine diagnosis was based on the International Classification of Headache Disorders (ICHD-3-beta) criteria (Headache Classification Committee of the International Headache Society (IHS) 2013). People with migraine headache preceded by visual aura in at least 30% of the attacks were included. They had to have at least 1 migraine attack per year, at least one in the year preceding the study and no more than eight attacks or 15 headache days per month (thus excluding chronic migraine). Those using prophylactic migraine medication were not included. Experimental sessions were performed at least 72 h after a migraine attack. Sessions that were followed by a migraine attack within 72 h, verified by follow-up, were excluded.

Participants with migraine were matched with controls based on age, gender and RMT. Matching on RMT was performed to correct for effects of stimulation intensity and thereby prevent possible differences in threshold between groups to confound TEP readouts. Only controls without a history of epilepsy or migraine were included. Participants (with migraine and controls) with contra-indication to TMS, pregnant women and people with diabetes mellitus, psychiatric conditions and people using medication that could affect cortical excitability (such as psychoactive drugs and beta-blockers) were excluded. We established that participants did not smoke, used drugs or drank alcohol or coffee in the 12 h preceding the measurement and to maintain a normal sleep pattern the night prior to the measurement. Written informed consent was obtained from all individual participants included in the study. The study was approved by Ethical Committee of Erasmus University Medical Centre, Rotterdam, the Netherlands, and conducted according to the Declaration of Helsinki.

### Recording Setup

#### Transcranial Magnetic Stimulation

TMS was performed with a MagPro X100 magnetic stimulator (Magventure, Denmark), a 14 cm diameter parabolic circular coil (type MMC-140) using biphasic pulses with a width of 280 µs, to activate a large region of the cortex, including the motor cortex (Kimiskidis et al. 2017), or a sham coil (type MCF-P-B65). Measurements were conducted between 09.00 AM and 04.00 PM and distributed evenly between AM and PM in both participant groups. Soft foam earplugs were used to dampen the TMS-induced coil click.

#### Electromyography

Motor evoked potentials were recorded bilaterally from the Abductor Pollicis Brevis muscles with a Nicolet Viking EDX electromyograph (Natus, Madison, WI, USA). Muscle activity was monitored using real-time visual feedback. Data were recorded with a sampling frequency of 4 kHz and stored for offline analysis.

#### Electroencephalography

EEG was recorded during the TMS sessions with a 64-channel TMS-compatible EEG system (Waveguard^™^ cap and ASAlab^™^ software, ANT Neuro, Enschede, The Netherlands), a sampling frequency of 4 kHz and a common average reference. Electrode impedance was kept below 5 kOhm during the experiment. Participants were seated in a comfortable chair with their eyes open and arms in supine position. Prior to stimulation, baseline EEG was recorded for 10 min with eyes open (5 min) and closed (5 min).

### Single Pulse TMS Protocol

To be suitable for clinical settings, the stimulation protocol we employed was designed to be short while yielding maximum information at once (Bauer et al. 2019). The stimulation procedure was performed using counterclockwise (right hemisphere) and clockwise (left hemisphere) stimulation. With the centre of the circular coil on electrode position Cz (vertex) the RMT, defined as lowest stimulation intensity evoking motor evoked potentials larger than 50 µV in 50% of the trials (Groppa et al. 2012), was determined. Then, a semi-automated, in-house designed stimulation protocol (created in Matlab® (version 2007b, The MathWorks, Natick, MA)) was used to deliver stimuli with a frequency of 0.5 Hz (Herring et al. 2015). Stimulation started at a stimulator output value of RMT minus 10% and increased in 2% steps until a reproducible motor evoked potential (> 200 µV) was seen after every stimulus (± 110–120% RMT). At each intensity 20 stimuli were given and aggregated for TEP analyses to limit the participant’s exposure to TMS stimuli. This stimulation procedure was repeated for the sham protocol using the sham coil, including the stepwise increments in stimulation intensity with matching intensities to the active coil.

### Data Analysis

#### Data Pre-Processing

Off-line analyses were performed in Matlab® (version 2015a) using custom-written scripts and the FieldTrip Matlab toolbox (Oostenveld et al. 2011). A TMS-EEG artefact removal pipeline (Herring et al. 2015) was used to eliminate ringing, decay, muscle and eye movement artefacts. Only trials performed at stimulation intensities between + 0% and + 6% stimulator output relative to the averaged RMT of two hemispheres were pooled and used for further analyses. All the datasets, both active and sham stimulation, were split in trial epochs starting 1 s before and ending 1 s after the TMS pulse. Ringing artefacts were segmented out from 0 to 6 ms relative to the time of stimulation and baseline corrected using the window from -200 ms to -1 ms relative to the start of the stimulus. Electrodes showing contaminated activity (e.g. excessive line noise) over the averaged trials were removed for each participant (average: 1 channel per participant, range: 0–4 channels). EEG data were then re-referenced to the common grand average of all non-interpolated EEG channels.

Next, independent component analysis (ICA) was used to remove exponential decay artefacts, recharge artefacts, eye blinks, eye movements and line noise for both the active coil and sham datasets. A maximum of 63 components were extracted from the data (number of components equal to the number of non-interpolated EEG channels minus 1), on average 8 components were removed in the first round of ICA (range: 3–18 components). The ICA decomposition was back-projected to the channel level after removal of the independent components containing the artefacts. Trial epochs were shortened to windows starting 200 ms before and ending 600 ms after the TMS pulse, followed by a second round of ICA to remove muscle related artefacts and remaining line noise artefacts (average of 8 components, range: 4–15). After reconstruction of the channel level data the split trials were re-combined. To completely remove residual time-locked muscle artefacts not captured by ICA, cubic interpolation was used from 1 ms to 15 ms around the stimulus. Next, some additional pre-processing steps were performed, dependent on the type of analysis (time-amplitude or time-frequency), as specified below.

#### Time-Amplitude Processing

Individual trials were baseline corrected and band-pass filtered between 1 and 80 Hz using a 3th order Butterworth filter. Removed electrodes were spherically interpolated. Trials were visually inspected and those which still showed contaminated activity were discarded. The resulting dataset consisted of 80 trials per current direction per participant (excluding removed trials). The TEP waveform was averaged over all trials and per current direction for each electrode. In addition, the global mean field power (GMFP), was calculated over both current directions and for each current direction separately (Esser et al. 2006).

#### Time-Frequency Processing

Frequency spectra and phase clustering index (Kalitzin et al. 2002) were calculated at all electrodes using Morlet wavelets. Three cycles/frequency were used for high temporal resolution, in 1 Hz frequency steps between 5 and 80 Hz and 5 ms time steps. To limit the number of comparisons (time-frequency versus time-frequency-electrode points) and to study in particular occipital responses in migraine with aura, the frequency spectra and phase clustering index were compared for three a priori defined frontal, central and occipital electrode clusters. Phase clustering index values vary between 0 (random phase clustering between trials) and 1 (all trials have equal phase clustering) per time-frequency point.

### Statistical Analysis

Magnetic or sham stimulation responses were compared between migraine and control groups for the window from 20 to 200 ms after stimulation (720 samples per channel at 4000 Hz sampling rate with a total of 62 channels). Within this time window the commonly reported TEP peaks are present across all channels (Suppl. Figure 2), allowing time-electrode cluster analysis of evoked activity. Statistical analysis was performed over all channels within the specified window for the combined dataset (with pooling of both current directions). To investigate consistency of the results, we also repeated the statistical analysis for each current direction separately.

TEPs were compared between groups using dependent *t*-tests (using the matched case-control design) at all samples within the pre-defined time window for the electrodes. In addition, we identified three regions of interest a priori: frontal (electrodes F1, F2, Fz, FCz, AF4, AF3), central (Cz, C1, C2, CPz, CP1, CP2) and occipital (Oz, O1, O2, POz, PO3, PO4), to limit the number of comparisons and to especially study occipital responses in migraine with aura. Exact *p*-values were calculated by enumeration using cluster-based permutation testing to correct for multiple comparisons and the small sample size (Maris and Oostenveld 2007) using the FieldTrip Matlab toolbox (Oostenveld et al. 2011). Clusters based on adjacency in time and electrode space were formed using samples with a cluster-alpha of 0.10 (independent *t*-test). This threshold allows for detection of larger clusters in the time-electrode space, without selection of separate clusters of single time-electrode points detected at *p* < 0.05 as a cut-off (Maris and Oostenveld 2007). Within each cluster, *t*-values (for both time samples and electrodes) were summed and compared to a dataset of all possible combinations of the original data (1024 combinations using the matched pair design). Clusters were considered significantly different between groups when their summed *t*-values where lower or higher than 2.5% (*p* < 0.025) of all permuted clusters.

## Results

Ten individuals with migraine were assessed (9 females, 1 male; mean age 41 years, range 21–62; 3 left-handed), who were also included in previous research (Bauer et al. 2019). The migraine attack frequency was between 0.3 and 2 per month (average of 0.9 attacks). Ten controls were included. Characteristics of participants, including sex, age, attack frequency and duration, are summarised in Table 1. Data from the individual participants with migraine are provided in Suppl. Table S1. All participants tolerated the experimental sessions. No migraine attacks were reported in the 72 h following the experiment.

**Table 1:** – Demographic, clinical and experimental data for healthy controls and migraine patients with aura reported as mean (± SD) or number

	Control	Migraine with aura
No. (female / male)	10 (9/1)	10 (9/1)
Age [years]	39.8 (± 11.1)	41.0 (± 12.6)
Age at onset [years]	-	17.8 (± 4.5)
Attack frequency [/month]	-	0.9 (± 0.6)
Mean headache duration [hrs]	-	25 (± 19)
Aura frequency [% of attacks]	-	86 (± 28)
RMT [% MSO]	41.1 (± 6.6)	41.3 (± 4.4)
Number of pulses	298 (± 29)	293 (± 35)
Removed ICA components	8.1 (± 2.7)	7.4 (± 1.9)

RMT: resting motor threshold; ICA: independent component analyses; MSO: maximum stimulator output

### Effect of TMS Current Direction

**Fig. 1:**
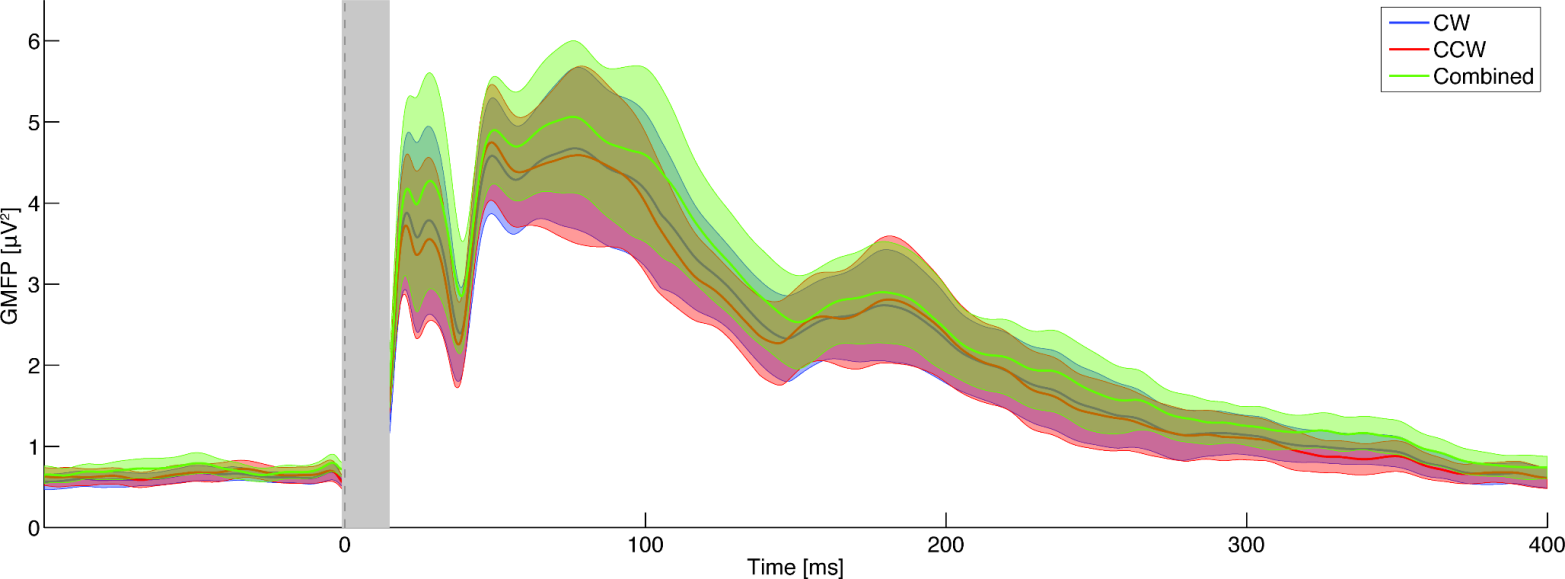
In controls and people with migraine the global mean field power (GMFP) of the average TEP responses show no waveform differences (i.e. direction and delay of the various peaks) with comparable peak distributions between clockwise (blue line) and counterclockwise (red line) current direction and when both current directions are combined (green line). Plot shows mean and patched standard error, the grey bar indicates the spherically interpolated parts of the EEG traces (-1 to 15 ms). CW: clockwise; CCW: counterclockwise

**Fig. 2:**
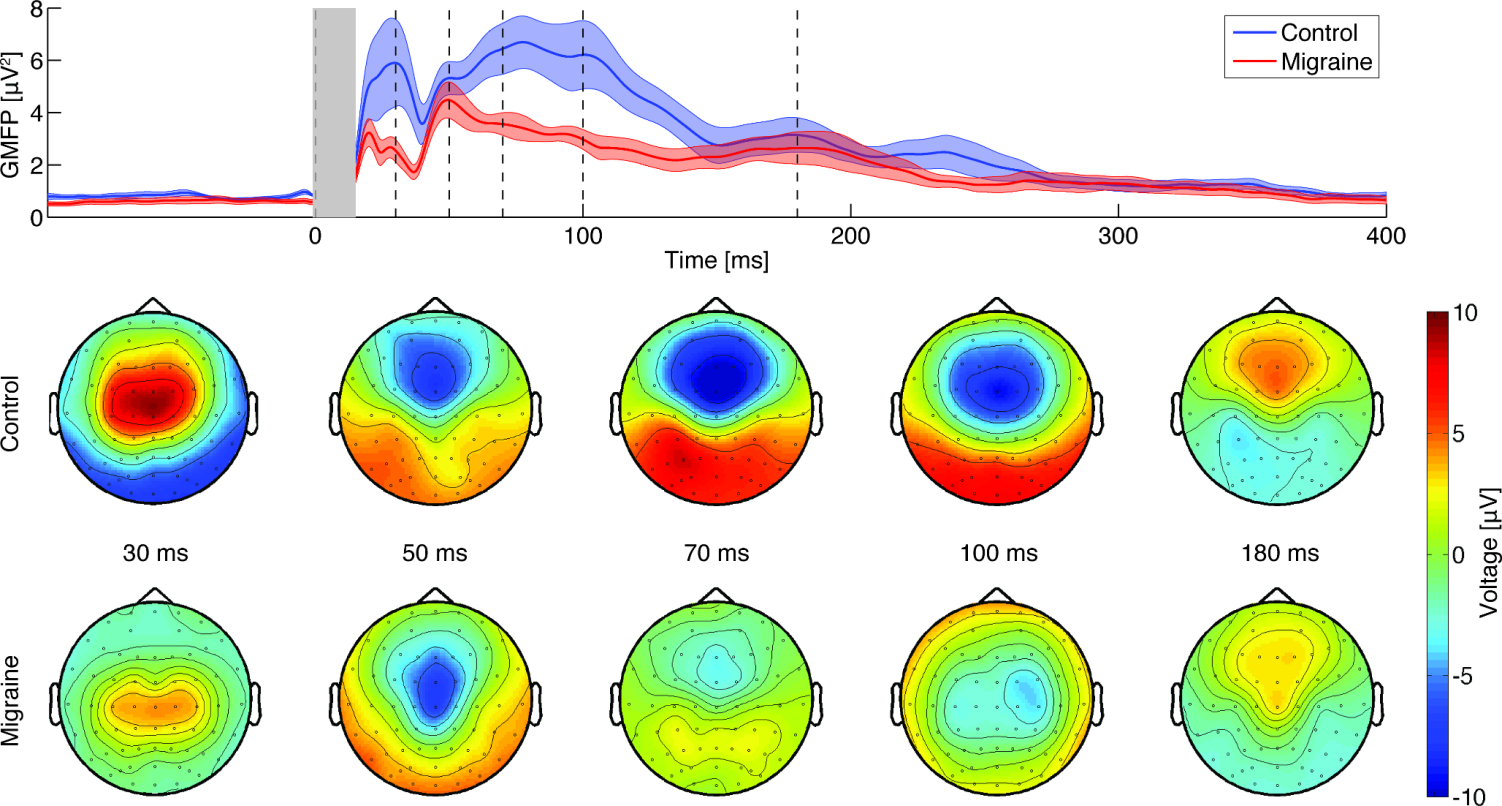
Comparison of the global mean field power (GMFP) of the TMS-evoked potentials of the combined clockwise and counterclockwise trials between control (blue) and migraine groups (red). Top plot shows mean and patched standard error, the grey bar indicates the spherically interpolated parts of the EEG traces (-1 to 15 ms) and dashed black lines the time corresponding to the topoplots. Bottom: the corresponding topographical plots for the P30, P50, P70, N100, and P180 peaks

First, possible differences between clockwise and counterclockwise stimulation trials were analysed within all subjects (60–80 trials per participant), combining migraine and control groups. The GMFP for both current directions and the combined dataset was computed (Fig. 1). Averaged TEP waveforms did not differ between polarities over time and electrodes for frontal (*p* = 0.28), central (*p* = 0.20), and occipital waveforms (*p* = 0.30), but differed when analysed over the entire scalp (*p* = 0.004; Suppl. Figure 1A). The difference clusters were present over primary motor and somatosensory cortices (Suppl. Figure 1B), likely due to the relationship between current direction and preferential activation (Rösler et al. 1989). Clockwise and counterclockwise trials were grouped for further analyses, and, as secondary outcome, also analysed per current direction. The GMFP and the corresponding topographical distributions are visualized in Fig. 2. Although the averaged TEP waveforms differed between current direction over the scalp in response to stimulation at Cz, they were of similar shape at the same electrode locations for migraine and control groups (Suppl. Figure 2).

### Sham Stimulation Evoked Potentials

Evoked responses induced by sham stimulation (averaged over 80 trials) showed a clear N100-P180 auditory complex (Suppl. Figures 3 and 4; ter Braack et al. 2015) in both healthy controls and participants with migraine. Averaged waveforms after sham stimulation did not differ between migraine and controls over time and all electrodes (*p* = 0.59) nor over the predefined electrode groups (all *p* > 0.28).

### TMS Evoked Responses

No significant differences were observed for the peak-to-peak amplitude analysis of the motor evoked potentials for people with migraine compared to their matched controls (Suppl. Table S2).

**Fig. 3:**
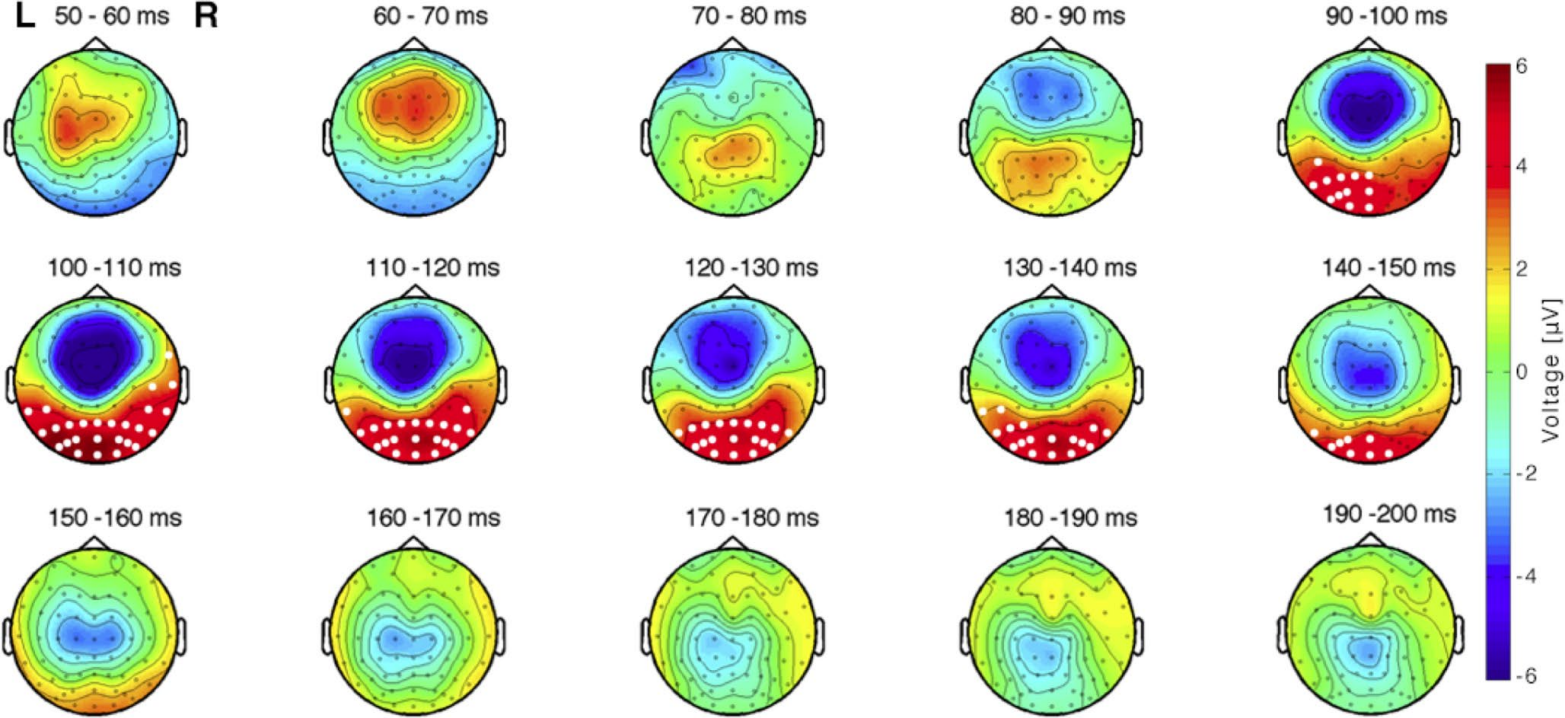
Topographical plots of difference in TEP amplitude between controls and migraine subjects show one distinct difference component. Plots display the averaged difference (control minus migraine) in 10-ms windows between 50 and 200 ms. Note that statistical analyses were carried out per ms; results were pooled in 10-ms bins for visualization purposes only. The significant cluster is highlighted over time with white dots at the significantly differing electrode positions, mainly located over the occipital cortex between 90 and 150 ms

**Fig. 4:**
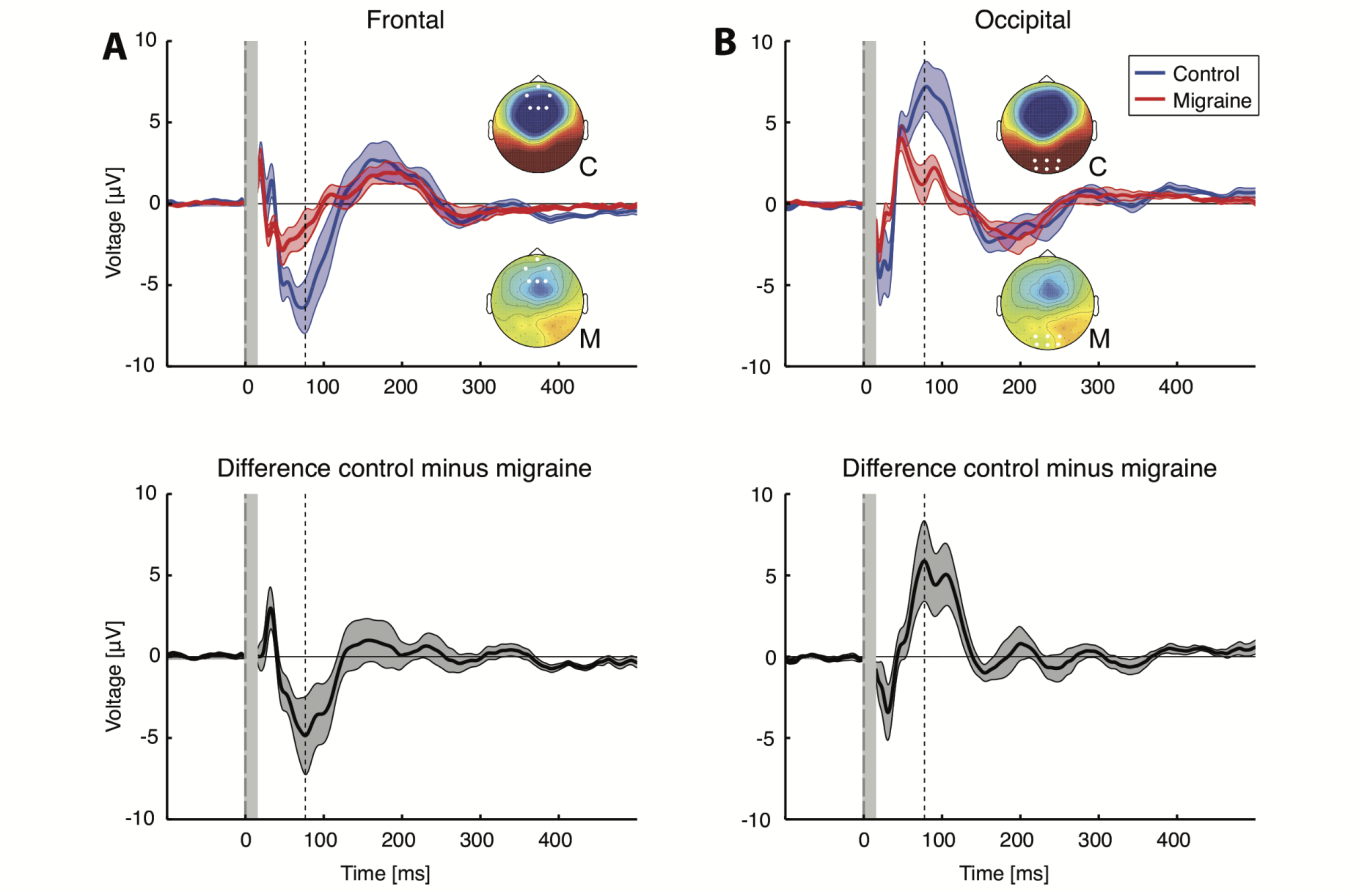
Grand-averaged TEP responses and difference waveform (control minus migraine) at frontal (F1, F2, Fz, Fpz, AF4, AF3) and occipital (Oz, O1, O2, POz, PO3 and PO4) electrodes show differences in TEP peaks between controls and migraine subjects. Two separate components of a negative waveform cluster were found using exact cluster-based permutation testing (enumeration). (**a**) Migraine group (red line) shows decreased frontal activity around the TEP N100 peak compared to control group (blue line), with largest difference of -4.9 µV at 77 ms after stimulation (dashed line). Bottom plot shows the difference between migraine and control groups (standard error of the mean calculated using 10.000 bootstraps over both groups). (**b**) Occipitally, the TEP N100 peak decreased as well in migraine, with largest difference of -5.9 µV at 78 ms after stimulation (dashed line). Bottom plot shows the difference between migraine and control groups. Insets show topographical distribution in control (C) and migraine (M) at the time point of maximal difference with electrodes highlighted in white dots. Traces show grand-averaged mean with patched standard error. The grey bars indicate the spherically interpolated parts of the EEG traces (-1 to 15 ms)

Cluster-based permutation analysis of TEP amplitudes over time and electrodes showed a significant difference in the a priori selected time interval between 20 and 200 ms after stimulation (*p* = 0.012 for combined polarities, *p* = 0.013 for CW stimulation and *p* = 0.018 for CCW stimulation) for people with migraine compared to controls. The revealed cluster was grouped around the N100 peak, between 60 and 120 ms after stimulation, and located mainly at the occipital cortex (Fig. 3). When analysed in the predefined electrode groups (frontal, central and occipital), no statistically significant difference was present at the central electrodes (*p*_*=*_0.050 for combined polarities, *p* = 0.060 for CW stimulation and *p* = 0.025 for CCW stimulation). The N100 peak, however, was smaller in the migraine group at the frontal electrodes (*p* = 0.009 for combined polarities, *p* = 0.019 for CW stimulation and *p* = 0.009 for CCW stimulation). The largest difference in the frontal cluster (4.9±0.9 µV) was present at 77 ms after stimulation (Fig. 4A). Also at the occipital cortex, the N100 peak was decreased in people with migraine compared to controls (*p* = 0.008 for combined polarities, *p* = 0.009 for CW stimulation and *p* = 0.005 for CCW stimulation). Here, the largest difference (5.9±0.9 µV) was found at 78 ms after stimulation, similar to the frontal cluster (Fig. 4B). The TEP P180 peak between 120 and 180 ms was not different for any of the electrode locations in people with migraine compared to controls, as no significantly different clusters were found.

*Post hoc* analysis of the TEP responses with sham responses subtracted revealed similar results (Suppl. Figure 5), albeit with slight differences in cluster sizes and their *p*-values. For the comparison over time and all electrodes we observed a significant difference in the a priori selected time interval between 20 and 200 ms after stimulation (*p* = 0.008 for a positive and *p* = 0.0167 for a negative cluster for combined polarities, a single positive cluster *p* = 0.0156 for CW and no significant cluster for CCW stimulation) for people with migraine compared to controls. The observed cluster was grouped around the N100 peak between 60 and 120 ms after stimulation and located mainly at the occipital cortex. When analysed in the predefined electrode groups (frontal, central and occipital), the N100 peak was smaller in the migraine group at the frontal cortex (*p* < 0.001 for combined polarities, *p* = 0.005 for CW stimulation and *p* = 0.041 for CCW stimulation) and occipital cortex (*p* = 0.009 for combined polarities, *p* = 0.012 for CW stimulation and *p* = 0.0016 for CCW stimulation) electrodes when compared to controls. For the central electrode cluster a significant difference was present only for the CCW stimulation (*p* = 0.035 for combined polarities, *p* = 0.038 for CW stimulation and *p* = 0.008 for CCW stimulation).

Time-frequency analyses between 20 and 200 ms and for the 5–80 Hz frequency bands, of spectral power and phase clustering over trials within the time-frequency domain, resulted in no significant clusters for any of the comparisons made. The statistics are reported in the Supplementary Results.

## Discussion

Our data show altered cortical EEG responses to transcranial magnetic stimulation in-between attacks in migraine with aura compared to controls. We demonstrated that TEP amplitude waveforms in migraine with aura are distinct from those in healthy controls, by displaying a reduced amplitude around the frontal and occipital N100 peak. No difference was observed between people with migraine and controls in the distribution of waveforms over the entire scalp. We cannot rule out, however, that the N100 amplitude reduction observed for migraine may (in part) reflect differences in sensory activation between groups that were not adequately compensated for by the utilized sham protocol without a sound masking procedure or electrical stimulation component. Our findings nevertheless suggest that TEP features could be suitable markers of cortical excitability changes in migraine. Alterations in cortical excitability over the migraine cycle, as indicated by indirect studies of brain excitability (Cosentino et al. 2014), could possibly be studied by longitudinal application of TMS-EEG.

Analyses of TEP waveforms showed two distinct regions in which the N100 amplitude responses were decreased in migraine with aura: (i) at the level of the frontal cortex, and (ii) at the level of the occipital cortex. Our finding of a *decreased* N100 peak may reflect decreased cortical inhibition at the level of the frontal and occipital cortex, since *increased* N100 peak amplitude has been indicated to reflect increased inhibitory GABA_B_ mediated receptor activation (Premoli et al. 2014; Rogasch et al. 2013). A larger N100 peak in epilepsy was attributed to increased activation of inhibitory circuits as a possible result of the use of anti-epileptic drugs, which could have enhanced GABA-ergic activity (ter Braack et al. 2016). The physiological underpinnings of various TEP peaks are, however, not straightforward (Conde et al. 2019; Raffin et al. 2020). While some studies report a linear dependency of the GABA_B_-ergic effect on N100 and P180 TEP peak amplitudes (Premoli et al. 2014; Casula et al. 2018), other studies only report a direct effect of GABA_B_ on the N100 peak, but not the P180 peak amplitude (Premoli et al. 2014; Rogasch et al. 2013).

The frontal cortex was suggested to play a role in controlling pain processing in migraine. Reduced EEG-based activation of the anterior-medial prefrontal cortices during contact-heat stimuli in migraine with aura was interpreted as a heightened state of readiness to anticipated pain, compared to controls (Lev et al. 2013). Also, the dorsolateral pre-frontal cortex (DLPFC) inhibits cortical as well as subcortical pain pathways (Lorenz et al. 2003). If decreased DLPFC cortical inhibition represents reduced DLPFC inhibitory *output*, it could contribute to enhanced pain perception in migraine. Alternatively, if decreased DLPFC cortical inhibition represents reduced intracortical inhibition *within* the DLPFC, this would be expected to result in an enhanced inhibitory output from the DLPFC on cortical and subcortical pain processing. This could be hypothesized to represent a protective mechanism against recurrent headaches in episodic migraine. Indeed, modulating DLPFC activity using high-frequency repetitive TMS decreased the number of monthly attacks in chronic migraine (Brighina et al. 2004). This suggests a role for the frontal cortex in migraine susceptibility, although the precise contribution of GABAergic inhibition remains unclear.

The observed decreased occipital TEP waveform around the N100 peak in migraine patients may also be explained by a decrease in cortical GABAergic inhibition, as indicated by TEP studies in healthy subjects (Premoli et al. 2014a). With repeated visual stimulation in migraine, a decrease in habituation was attributed to lateral inhibitory processes in the thalamocortical network that was suggested to be mediated by GABAergic neurons in the occipital cortex (Coppola et al. 2013). Preclinically, single pulse TMS applied to the occipital cortex in rodents increased the threshold for inducing cortical spreading depolarization, the neurobiological correlate of the migraine aura, in the visual cortex (Lloyd et al. 2020). GABA_A/B_ antagonists reversed this effect, which indicates that TMS can suppress cortical neuronal activity by influencing GABAergic circuits (Lloyd et al. 2020). Paired pulse TMS to study short-interval intracortical inhibition (Cash et al. 2017) could be used to further investigate the role of GABAergic networks in altered cortical responsivity in migraine.

A decreased N100 peak was related to disrupted phase coherence in people with Huntington’s Disease (Casula et al. 2018). We found no altered phase clustering in people with migraine while the TEP N100 amplitude was decreased compared to controls. Our approach of full TEP waveform analyses instead of peak amplitude extraction, however, limits a direct comparison. In future studies, the electrode clusters and time windows of interest as revealed by our exploratory approach could be used to further explore the relationship between TEP amplitude and phase coherence in migraine.

A critical limitation of our study is the concern that the EEG response to TMS-related sound and sensory activation may differ between people with migraine and controls. TEP N100 and P180 peaks have been associated with auditory evoked responses (ter Braack et al. 2015) and somatosensory activation (Conde et al. 2019). With realistic sham stimulation at different locations on the scalp, activation patterns similar to TEPs have been measured with prominent N100 and P180 peaks (Conde et al. 2019; Gordon et al. 2021; Rocchi et al. 2021). Especially the N100 peak has been related to cortical excitability using direct intervention with benzodiazepines (Premoli et al. 2014a), in line with our finding that the P180 peak was not changed in people with migraine. As sensory processing of different modalities, including differences in the processing of auditory stimuli, appears altered in migraine (de Tommaso et al. 2014), the sound of the coil click during stimulation could partially explain observed differences in the TEP N100 response. We controlled for auditory and vibration-related effects of TMS by using sham stimulation, which produces a coil click and mechanical vibrations matched to those of the active coil. Furthermore, all participants wore soft foam earplugs during real and sham stimulation. In our *post hoc* analyses we subtracted sham waveforms from the TEP, assuming a linear interaction between active and sham responses. Similar results were obtained as with the TEP based analyses, indicating that differences between migraine and control groups in our study may reflect alterations in cortical excitability, but could also be due to differences in sensory activation not adequately compensated for by the used sham protocol. A linear subtraction, however, is limited in its application as the brain is a complex dynamical system with many nonlinear interactions, also between different types of somatosensory stimulation (Gordon et al. 2021, Chowdhurry et al. 2022). Modern sham stimulation, including an electric stimulation component to evoke somatosensory evoked potentials, shows activation patterns highly similar to TEPs with prominent N100 and P180 peaks (Gordon et al. 2021; Rocchi et al. 2021), indicating that those peaks are at least partially generated by somatosensory and/or auditory potentials. Those studies used below RMT stimulation intensities to limit the impact of sensory re-afferents, which as a trade-off may have limited the amplitude and phase locking of any late evoked cortical potentials. This contrasts with our work where we utilized stimulation intensities around and above RMT (i.e. ranging from + 0% to + 6% stimulator output relative to RMT) without active noise masking (i.e. only foam ear-plugs) and observed N100 peaks with much higher amplitudes when compared to the sham evoked potentials. Considering that we observed no differences in evoked motor responses (Suppl. Table S2) and that the location of the observed N100 cluster differed from those of the CW versus CCW comparison, we consider it unlikely, but cannot rule out, that the observed results are attributable to differences in processing of the motor responses by sensory re-afferents of peripheral muscles. Another group explored the input-output curves of TEPs, ranging from 20% RMT to 120% RMT, using modern sham with an electrical stimulation component (Raffin et al. 2020). They observed TEP waveforms with region-specific profiles depending on stimulation location, including differences in the waveforms of the late components at higher stimulation intensities. The interpretation of TMS evoked potentials and the origin of peaks is thus anything but straightforward and remains difficult and ambiguous. Future studies can address the contribution of auditory and sensory components to the TMS-evoked response features in migraine by using a realistic sham stimulation, such as synchronous sound masking and an electrical stimulation component for masking skin sensation (Gordon et al. 2021; Grasin et al. 2019).

Besides the used sham procedure, there are additional methodological limitations and considerations. Firstly, to improve artefact removal using independent component analysis, we combined trials at suprathreshold stimulation intensities and both current directions. The signal-to-noise ratio of our waveforms, frequency spectra and phase clustering readouts also benefitted from the larger number of trials. The pooling of trials at multiple stimulation intensities shortens the stimulation protocol (Bauer et al. 2019) and is supported by the relatively similar TEP waveforms in the small range of stimulation intensities, between 100 and 110% of RMT (Komssi et al. 2004). The within-subject comparison of the effect of current direction over all electrodes revealed significant clusters located over the centroparietal electrodes corresponding to the primary and somatosensory motor cortex, probably due to the preferential activation of a hemisphere with clockwise and counterclockwise current direction (Rösler et al. 1989). Comparison of the frontal, central and occipital electrode clusters, however, revealed no significant difference between current directions. We, therefore, used the combined trials for the primary endpoints in the group comparisons. The independence of our results from the used current direction was demonstrated by the separate analyses per current direction, which showed no differences to the results for the combined trials.

Secondly, we used non-focal stimulation over the vertex using a circular coil to achieve diffuse activation of the cortex. This approach has been utilized to investigate the widely distributed epileptogenic networks of genetic generalized epilepsy by using high intensity stimuli to provoke epileptiform discharges (Kimiskidis et al. 2017). In the context of our explorative study we considered the use of a circular coil most appropriate to induce broad cortical activations, without limiting the measurement to a pre-defined stimulation region of interest with a focal figure-of-eight coil. This allowed comparison of responses in various cortical regions, despite limiting the physiological interpretation of our findings. TEP waveforms induced by circular coil stimulation have been shown to be similar to focally induced waveforms in research with figure-of-eight coils (e.g. Conde et al. 2019, Premoli et al. 2014). Localization of responses was limited to their scalp distribution, as we have not implemented source localization. In future studies, probing the here identified regions, i.e. the frontal and occipital cortices, with focal stimulation with similar readouts would be a way to verify the present findings.

Thirdly, we cannot exclude a possible neuromodulatory effect of the repeated stimulation procedure. Stimuli were not jittered in this study because the stimulation protocol was specifically adapted to allow phase clustering analysis (Bauer et al. 2019). Using a non-jittered stimulation protocol we hypothesized to see differences in entrainment around the occipital cortex (Herring et al. 2015), however, no significant clusters across electrodes or between groups, were observed. The number of stimuli (at maximum 160 per direction) and stimulation frequency (0.5 Hz), was based on TMS-EEG literature where no neuromodulatory effects were reported (Farzan et al. 2010; Herring et al. 2015; ter Braack et al. 2015). A much more elaborate stimulation of 1200 stimuli presented at 1 Hz over the motor cortex in healthy controls revealed a regional inhibitory effect of prolonged stimulation, limited to the motor cortex and not affecting the visual cortex (Casula et al. 2014). The differences between migraine and control groups reported here are therefore unlikely to result from neuromodulatory effects due to prolonged single pulse TMS. Attention to auditory stimuli may be altered in migraine patients and attending to upcoming stimuli in a sequence can alter the response to a mixture of cortical and sensory stimulation (Masson et al. 2020). We did not observe significant clusters in the analysis of the sham measurements, indicating that an auditory-driven attention effect likely was minimal within our study population.

Lastly, our exploratory study is limited by a small sample size. To increase comparability between groups, we matched the subjects with migraine to healthy controls based on age, sex, and RMT. Matching cases and controls on RMT is not a standard approach, but we believe that this reduces the possibility of bias. The stimulation intensity was based on the RMT and matching on RMT ensures that the stimulation intensity was comparable for both groups and diminishes the effect of high RMT inter-individual variance (Kimiskidis et al. 2004; Koski et al. 2005) on our readouts. Matching based on RMT resulted in similar variance in both groups, but we cannot exclude a possible effect of the migraine or menstrual cycle on RMT variance (Cortese et al. 2017). We did not collect data about the menstrual cycle in our study. The limited number of studies assessing TMS-based cortical excitability measures in relation to the menstrual cycle indicate that cortical excitability might be unrelated from the menstrual hormone status in migraine (Boros et al. 2009), and in epilepsy (Badawy et al. 2013). We used exact permutation-based tests by enumeration, an approach known to remain robust with relatively small sample sizes (Maris and Oostenveld 2007). To increase the robustness of our statistical results, we compared the exact enumeration statistics with Monte Carlo permutation tests, which yielded similar results. Instead of performing peak-only analyses, our analyses were strengthened by analyzing the data for differences over time-electrode clusters (for TEPs). The finding of statistically significant differences in frontal and occipital TEP N100 amplitudes, despite the small number of study participants, indicates robust results with a large effect and only little inter-individual variation. Still, generalizability of our findings to the general migraine population may be limited due to the sample size and by the inclusion of only participants with migraine with aura. Future studies including larger numbers of participants with migraine with and without aura should therefore determine the reproducibility and generalizability of our observations.

In conclusion, people with migraine with aura show distinct cortical EEG responses to magnetic stimulation compared to controls in the periods in-between attacks. The observed peak amplitude differences suggest a possible reduction in cortical inhibition in migraine, but alternatively they could also reflect changes in sensory activation between groups, or involve both mechanisms. Our findings are in line with reports of altered interictal cortical excitability in migraine that were based on indirect measures, using e.g. visual or somatosensory inputs, or magnetic stimulation with peripheral readouts. In our study, all participants tolerated the TMS-EEG experimental procedure well and no induced migraine attacks were reported. This opens up possibilities for follow-up TMS studies in subjects without aura or with exclusive aura, studies exploring the correlation between clinical parameters (such as attack frequency or duration) and TEP peak amplitudes, and for longitudinal TMS-EEG studies during the migraine cycle. Such studies could strengthen the specificity of our findings for migraine with aura, and provide insight in changes of cortical excitability related to the onset of a migraine attack.

## Electronic Supplementary Material

Below is the link to the electronic supplementary material.

Supplementary Material 1

## Data Availability

Data will be made available upon reasonable request.
