# Supplementary material for: TMS-evoked EEG potentials demonstrate altered cortical excitability in migraine with aura
Source: Brain Topogr. 2023 Feb 13;36(2):269–81. doi: 10.1007/s10548-023-00943-2 (PMC10014725; doi:10.1007/s10548-023-00943-2)
Supplement: Supplementary file 1 — Supplementary Material 1 [file 10548_2023_943_MOESM1_ESM.pdf]

Supplementary material

TMS-evoked EEG potentials demonstrate altered cortical excitability in migraine with aura

Robert M Helling*, Matthijs JL Perenboom*, Prisca R Bauer, Johannes A Carpay, Josemir W Sander, Michel D Ferrari, Gerhard H Visser, Else A Tolner

Corresponding author: Else A. Tolner, PhD; Departments of Neurology & Human Genetics; Leiden University Medical Center; Email: E.A.Tolner@lumc.nl

Journal: Brain Topography

Supplementary Results

Time-frequency spectra

To assess differences in time-frequency power of TEPs between migraine and controls, cluster-based permutation analyses were conducted for the time-frequency spectra of the averaged responses (using wavelet analysis between 20-200 ms, and 5-80 Hz) for frontal, central, and occipital regions. No differences in time-frequency spectra were found in any of the predefined electrode clusters: (frontal p=0.09 (combined polarities), p=0.29 (CCW), p=0.04 (CCW)), central (p=0.12, p=0.34, p=0.08) nor occipital (p=0.29, p=0.35, p=0.11).

Phase clustering over trials

Consistency of TEP responses over trials was compared between groups using phase clustering analyses in the time-frequency domain. Statistical cluster-based permutation analyses were conducted for phase clustering over trials within the time-frequency domain over frontal, central and occipital electrode groups. There were no differences in phase clustering in migraine compared to controls, for none of the electrode groups and irrespective of current direction (frontal electrodes p=0.17 (combined polarities), p=0.33 (CCW), p=0.13 (CCW); central electrodes p=0.23, p=0.11, p=0.47; occipital electrodes p=0.17, p=0.089, p=0.18).

Time-frequency analyses of sham results

Analysing the sham dataset, again frequency spectra were not different between groups for the electrode clusters (all p>0.13). Phase clustering over trials did not include significantly different time-frequency clusters for the three electrode groups (all p>0.23).

Table S1. Individual patients data

Subject	M/F	Age at inclusion	Disease duration (years)	Attack frequency (events/month)	Percentage with aura	Attack duration (hours)
M01	M	50	45	1	100	12
M02	F	27	12	0,3	90	34
M03	F	48	12,5	0,5	100	0
M04	F	21	2	0,3	100	24
M05	F	45	32	1	100	34
M06	F	35	13	0,5	30	6
M07	F	40	15	2	100	24
M08	F	62	45	0,5	100	34
M09	F	51	33	1	100	72
M10	F	31	20	1,5	35	13,5

Table S2. Motor evoked potential (MEP) peak-to-peak amplitude (between 15-50 ms after stimulation) statistics for both groups, for the clockwise (CW), counterclockwise (CCW), combined current directions, and the comparison of the lowest RMT hemisphere and the corresponding contralateral hand motor responses only.

**Comparison**	**MEP peak-to-peak amplitude**		***p*-value**
	**Controls Mean (std)**	**Migraine Mean (std)**	
CW	108 (94) μV	118 (127) μV	0.86
CCW	117 (63) μV	92 (106) μV	0.51
Combined	112 (78) μV	105 (115) μV	0.84
Lowest RMT	156 (86) μV	145 (126) μV	0.83

Supplementary Figures

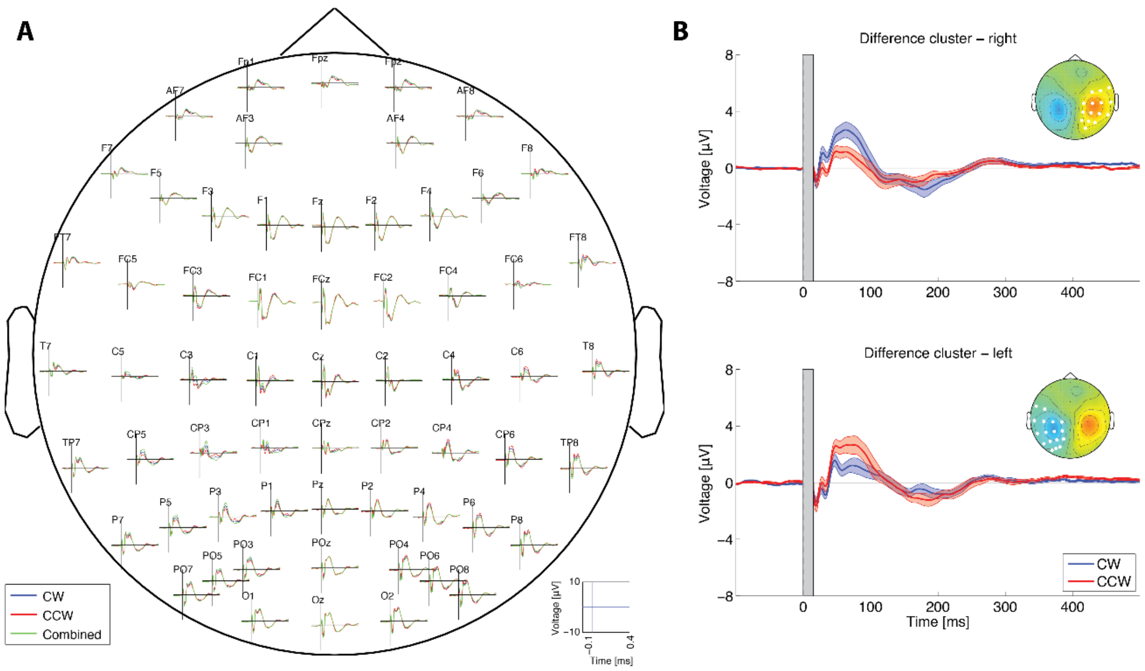

Fig. S1. (**a**) Distribution of average TEP waveforms over the scalp for clockwise (CW), counterclockwise (CCW) and combined polarities, averaged over all participants. Note the similarities in waveform between current directions (e.g. direction and delay of the N100 and P180 peaks). (**b**) Waveforms differ between CW and CCW stimulation over the primary and somatosensory motor cortices (per plot, the average waveform over the indicated electrodes is shown). The side of the difference depends on the current direction, i.e., CW stimulation evoking strongest response in the right hemisphere, and CCW stimulation evoking strongest response in the left hemisphere. Inserts show topoplots of the TEP difference waveform (CW minus CCW) distribution averaged between 70-80 ms after stimulation, where the mirrored activation between hemispheres is clearly visible. White dots display electrodes within the significantly different clusters, which are also mirrored between hemispheres depending on current direction.

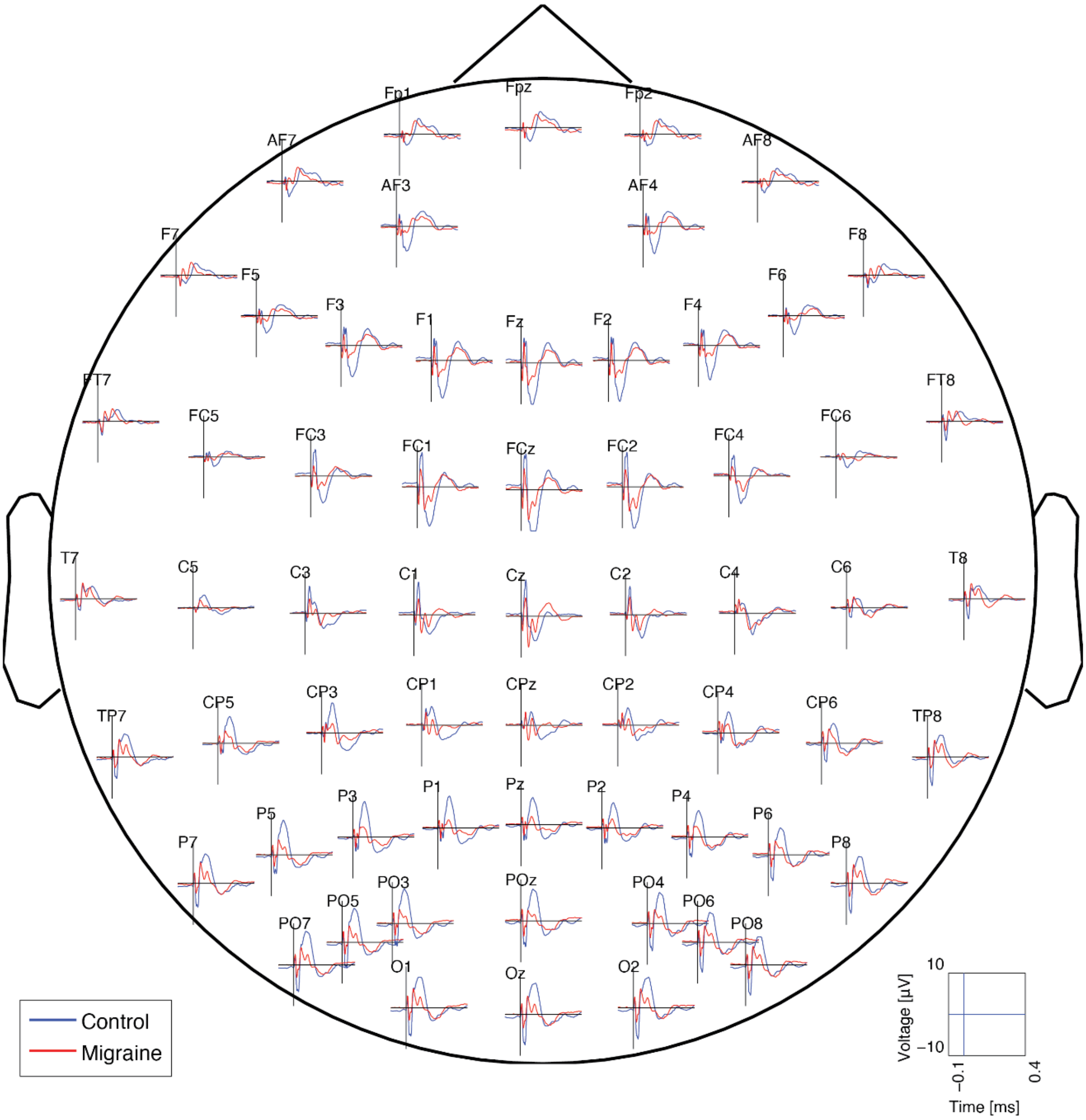

Fig. S2. Distribution of average TEP waveforms over the scalp for control (blue) and migraine groups (red). Note the similarities in waveform between groups (e.g. direction and delay of the N100 and P180 peaks).

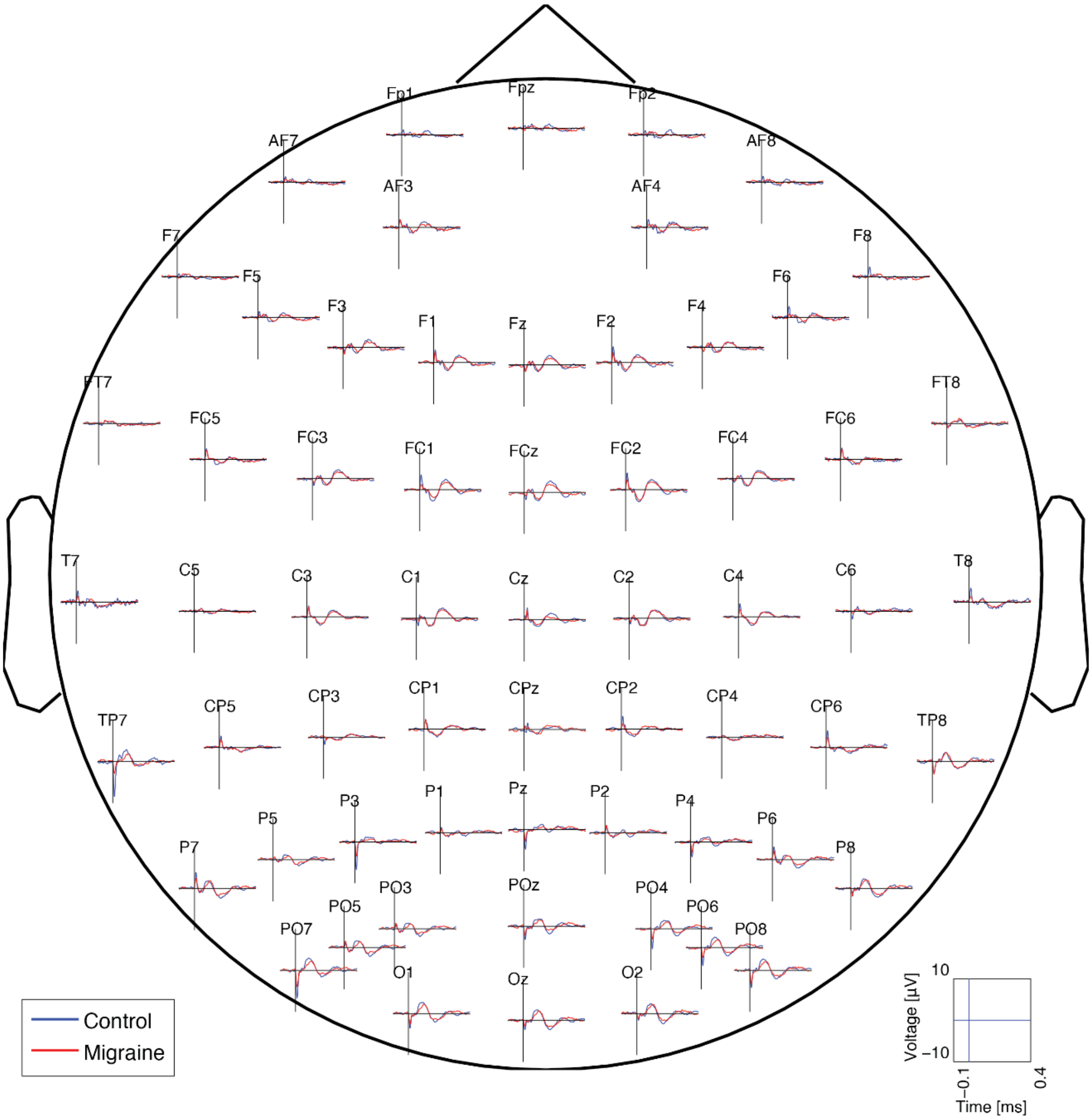

Fig. S3. Distribution of sham waveforms over the scalp for control (blue) and migraine (red) groups. Amplitude of the sham waveforms is much smaller compared to TEP waveforms (same y-axis limits are used as in Figure S2). Note the similarities in waveform between groups, like direction and delay of the sham-coil induced peaks around 100 and 180 ms.

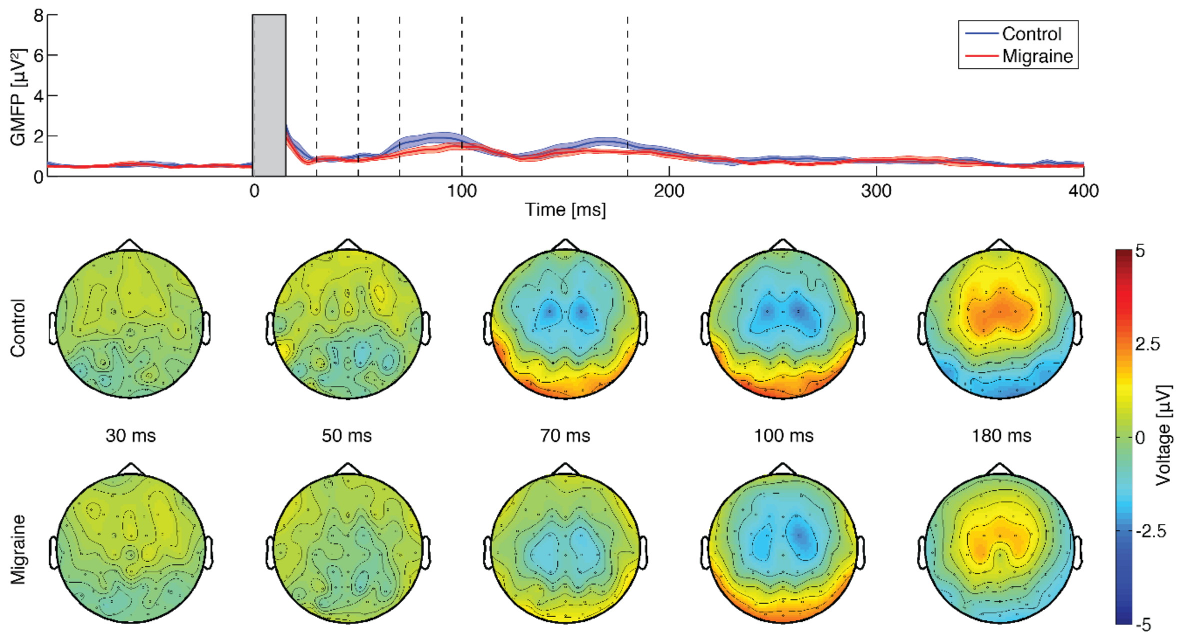

Fig. S4. Comparison of the global mean field power (GMFP) of the sham measurements between control (blue) and migraine groups (red). Top plot shows mean and patched standard error, the grey bar indicates the spherically interpolated parts of the EEG traces (-1 to 15 ms) and dashed black lines the time corresponding to the topoplots. Bottom: the corresponding topographical plots for the P30, P50, P70, N100, and P180 peaks.

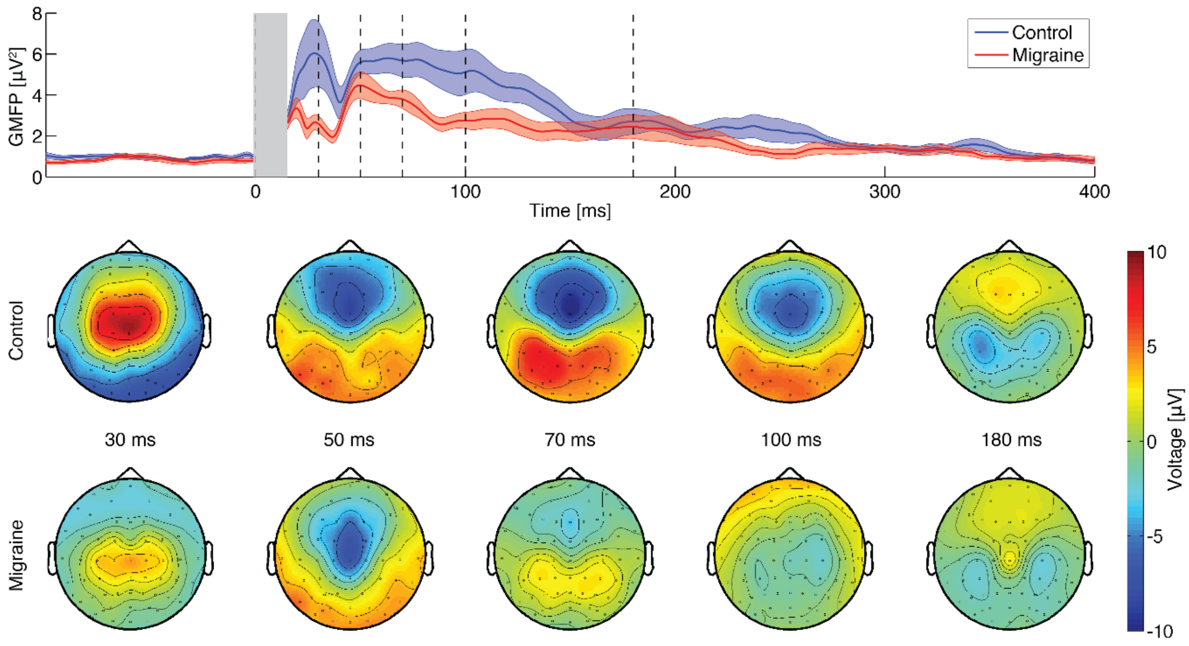

Fig. S5. Comparison between control (blue) and migraine groups (red) of the global mean field power (GMFP) of the TMS-evoked potentials (combined clockwise and counterclockwise trials) with the sham-evoked potentials linearly subtracted. Top plot shows mean and patched standard error, the grey bar indicates the spherically interpolated parts of the EEG traces (-1 to 15 ms) and dashed black lines the time corresponding to the topoplots. Bottom: the corresponding topographical plots for the P30, P50, P70, N100, and P180 peaks.
